# Knockdown of HSP110 attenuates hypoxia-induced pulmonary hypertension in mice through suppression of YAP/TAZ-TEAD4 pathway

**DOI:** 10.1186/s12931-022-02124-4

**Published:** 2022-08-19

**Authors:** Haitao Liu, Sha Zhang, Yi Liu, Jing Ma, Wei Chen, Tao Yin, Tongbin Li, Bin Liang, Ling Tao

**Affiliations:** 1 Department of Cardiology, The First Affiliated Hospital of Air Force Medical University, No. 15, Changle West Road, Xi’an, Shaanxi China; 2 Department of Traditional Chinese Medicine, The First Affiliated Hospital of Air Force Medical University, Xi’an, Shaanxi China; 3 Department of Cardiology, The Second Hospital of Shanxi Medical University, No. 382, Wuyi Road, Taiyuan, Shanxi China

**Keywords:** Pulmonary hypertension, Heat shock protein-110, Yes-associated protein, TEA domain transcription factor 4, Proliferation, Autophagy

## Abstract

**Background:**

Pulmonary hypertension (PH) is a progressive and fatal cardiopulmonary disease characterized by pulmonary vascular remodeling and increased pulmonary vascular resistance and artery pressure. Vascular remodeling is associated with the excessive cell proliferation and migration of pulmonary artery smooth muscle cells (PASMCs). In this paper, the effects of heat shock protein-110 (HSP110) on PH were investigated.

**Methods:**

The C57BL/6 mice and human PASMCs (HPASMCs) were respectively exposed to hypoxia to establish and simulate PH model in vivo and cell experiment in vitro. To HSP110 knockdown, the hypoxia mice and HPASMCs were infected with adeno-associated virus or adenovirus carring the shRNAs (short hairpin RNAs) for HSP110 (shHSP110). For HSP110 and yes-associated protein (YAP) overexpression, HPASMCs were infected with adenovirus vector carring the cDNA of HSP110 or YAP. The effects of HSP110 on PH development in mice and cell proliferation, migration and autophagy of PASMCs under hypoxia were assessed. Moreover, the regulatory mechanisms among HSP110, YAP and TEA domain transcription factor 4 (TEAD4) were investigated.

**Results:**

We demonstrated that expression of HSP110 was significantly increased in the pulmonary arteries of mice and HPASMCs under hypoxia. Moreover, knockdown of HSP110 alleviated hypoxia-induced right ventricle systolic pressure, vascular wall thickening, right ventricular hypertrophy, autophagy and proliferation of PASMCs in mice. In addition, knockdown of HSP110 inhibited the increases of proliferation, migration and autophagy of HPASMCs that induced by hypoxia in vitro. Mechanistically, HSP110 knockdown inhibited YAP and transcriptional co-activator with PDZ-binding motif (TAZ) activity and TEAD4 nuclear expression under hypoxia. However, overexpression of HSP110 exhibited the opposite results in HPASMCs. Additionally, overexpression of YAP partially restored the effects of shHSP110 on HPASMCs. The interaction of HSP110 and YAP was verified. Moreover, TEAD4 could promote the transcriptional activity of HSP110 by binding to the HSP110 promoter under hypoxia.

**Conclusions:**

Our findings suggest that HSP110 might contribute to the development of PH by regulating the proliferation, migration and autophagy of PASMCs through YAP/TAZ-TEAD4 pathway, which may help to understand deeper the pathogenic mechanism in PH development.

## Introduction

Pulmonary hypertension (PH) is a progressive and fatal cardiopulmonary disease characterized by pulmonary vascular remodeling and increased pulmonary vascular resistance and artery pressure, which eventually leads to right heart failure and death [1, 2]. A key pathological feature of PH is severe pulmonary vascular remodeling which could increase pulmonary artery pressure and vascular resistance [1, 3]. The excessive cell proliferation, apoptosis resistance and abnormal contractile-to-synthetic phenotype switching of pulmonary artery smooth muscle cells (PASMCs) are the main contributions to pulmonary vascular remodeling [3, 4]. It has proved by many studies that chronic hypoxia is a crucial factor contributing to PH by promoting the proliferation and migration of PASMCs in animal model [5, 6]. In this study, hypoxia induction was used to establish PH model in mice and cell culture. Autophagy is a conserved biological process for the degradation and recycling of damaged organelles and proteins in cells by lysosomes [7, 8]. Therefore, autophagy plays an important role in maintaining cell homeostasis and affecting human diseases. Many pathological stimulations such as hypoxia, starvation, cell damage could activate autophagy to exhibit a pro-survival role in damaged cells [9]. A large number of evidences have proved that autophagy induction is involved in the development of PH in rodent model and hypoxia-induced autophagy promotes the cell proliferation and migration of PASMCs, which leads to aggravate pulmonary vascular remodeling in PH model [10, 11].

Heat shock protein-110 (HSP110, also known as HSP105 or HSPH1) is a chaperone with anti-aggregation properties. HSP110 is able to interact with HSP70 to correct the folding of newly synthesized or misfolded proteins, which is important for protein homeostasis [12]. HSP110 also has other functions and it is involved in many human diseases. Studies found that HSP110 is upregulated in post-ischemic cortex in focal ischemia in rat, and the infarct volume and neurological deficit scores are decreased in HSP110 knockout (KO) mice [13, 14]. In addition, in lipopolysaccharide (LPS)-induced acute lung injury rat model, inhibition of HSP110 could alleviate lung injury through suppressing phosphorylation of signal transducer and activator of transcription 3 (STAT3) [15]. Moreover, HSP110 is highly expressed in many types of tumor such as gastric cancer, lung adenocarcinoma, colorectal cancer and B cell lymphoma and HSP110 possibly regulates the cell proliferation of these cancer cells [16–19]. According to gene expression profiling that analyzed by microarray assay from GEO database (https://www.ncbi.nlm.nih.gov/gds/, GSE113439), we found that the expression of HSP110 (HSPH1) was significantly increased in lung tissue from patients with PH compared to normal controls. These findings together suggest that HSP110 expression might be induced in PH and promote the cell proliferation of PASMCs in PH.

The yes-associated protein (YAP) and its paralog transcriptional co-activator with PDZ-binding motif (TAZ) are transcriptional modifiers and nuclear mechanotransducers. YAP/TAZ as transcriptional cofactors interact with TEA domain (TEAD) transcription factor family members including TEAD4 to form a complex to regulate target gene transcription [20–22]. For instance, Fan et al. reported that knockdown of YAP inhibited the proliferation, migration and angiogenesis of hypoxia-induced human umbilical vein endothelial cells (HUVECs) and it is probably because of forming a complex with TEAD4 to regulate transcription of genes that involved in cellular proliferation and angiogenesis [23]. Moreover, the crucial roles in development of vascular disease and pulmonary hypertension of YAP and TAZ have been revealed [24, 25]. YAP/TAZ forms a feedback circuit with microRNA-130/301 to promote vascular extracellular matrix remodeling and stiffening, which leads to increase pulmonary vascular cell crosstalk and vascular cell proliferation [26]. YAP/TAZ also promotes to cell proliferation by activating glutaminolysis in pulmonary artery endothelial cells (PAECs) and PASMCs to promote PH [27]. Moreover, inactivation of large tumor suppressor 1 (LATS1) activates YAP/TAZ and then increases the proliferation of PASMCs to aggravate pulmonary vascular remodeling in PH [28]. Therefore, it indicates YAP/TAZ plays an important role in PH development. In this work, we predicted the interaction between YAP and HSP110 using HitPredict database (http://www.hitpredict.org/) and the potential TEAD4 binding sits on HSP110 promoter using JASPAR database (http://jaspar.genereg.net/). Therefore, we put forward a hypothesis that HSP110 might promote cell proliferation, migration of PASMCs by promoting YAP/TAZ-TEAD4 activity and its transcription also might be regulated by TEAD4. Additionally, since some evidences proved that autophagy could be induced by YAP and TAZ [29, 30]. In this study, the effect of HSP110 on autophagy of PASMCs was investigated.

## Materials and methods

### Animal model

Eight-week-old mice (C57BL/6) were used in the experiments. Mice were housed in optimal conditions and fed standard chow and water ad libitum. Firstly, mice were randomly divided into normoxia group and hypoxia group. The mice in normoxia group were housed at 21% O_2_ and mice in hypoxia group were housed at 10% O_2_ for 4 weeks, respectively. In order to investigate the effects of HSP110 on hypoxia-induced PH, mice were randomly divided five groups: normoxia group, hypoxia group, hypoxia-shNC (short hairpin RNA of negative control) group, hypoxia-shHSP110-1 (shRNA-1 for HSP110) group and hypoxia-shHSP110-2 (shRNA-2 for HSP110) group. Animals from hypoxia-shNC, hypoxia-shHSP110-1 and hypoxia-shHSP110-2 group respectively received a tail vein injection of 1.0 × 10^11^ vector genomes (vg)/ml adeno-associated virus carring shNC, shHSP110-1 and shHSP110-2. Normoxia group and hypoxia group received a tail vein injection of PBS. Three days after injection, all mice from four hypoxia groups were housed at 10% O_2_ for 4 weeks. Mice were anesthetized and the right ventricle systolic pressure (RVSP) was measured. The lungs and hearts were excised for the further studies. The right ventricle (RV) was separated from left ventricular (LV) and interventricular septum (S), and then RV and (LV + S) severally weighed. The right ventricular hypertrophy was determined by the ratio of the weight of RV to the LV plus S [RV/(LV + S)].

### Histological analysis

Mice lung tissues were fixed, embedded in paraffin, and cut into 5-µm-thick sections. The sections were stained with hematoxylin and eosin (H&E) and then observed using a microscope (Olumpus, Japan). The ratio of pulmonary arterial medial thickness to total vessel size (media/CSA) was quantified.

### Cell culture and infection

Human PASMCs (HPASMCs) were purchased from icellbioscience (USA) and cultured in PriMed-iCELL-004-LS medium (icellbioscience) in a humidified 5% CO_2_ at 37 °C. For the hypoxic induction, HPASMCs were starved for 12 h without serum and then exposed to hypoxic condition of 2% O_2_, 93% N_2_ and 5% CO_2_ for 24 h. For cell proliferation assay, the cells were culture in hypoxic condition for 0, 12, 24 and 48 h before detection. For cell infection, HPASMCs were transduced with adenoviral packaging with adenovirus carring shRNAs targeting HSP110 (shHSP110-1 and shHSP110-2) or plasmid vector containing cDNA of YAP or HSP110 for 48 h. Real-time PCR (RT-PCR) and western blot were used to confirm the knockdown of HSP110 and overexpression of YAP or HSP110. Then the cells were used for hypoxic induction and other further experiments.

### Cell proliferation

Cell proliferation was determined by CCK-8 assay. The HPASMCs (5 × 10^3^) were seeded into 96-well plate. The cells were subjected to different procedures according to the different experiments. Then cell viability was detected by CCK-8 kit (Sigma, USA) at 0, 12, 24 and 48 h. The absorbance of each well at 450 nm was measured by a microplate reader (Biotek, USA).

### Wound healing assay

The cell migration was determined by wound healing assay. Briefly, the cells were seeded and cultured until cells covered the plate bottom, 1 μg/ml mitomycin C (Sigma) were treated to each well for 1 h, then cells were wounded using 200 μl pipette tip and cultured with serum free medium under normoxia or hypoxia. The wound images were captured at 0 and 24 h using a microscope (Olympus, Japan).

### RNA isolation and RT-PCR

To investigate the mRNA expression of HSP110 and YAP, the total RNA was isolated from tissues and cultured HPASMCs according to the RNApure total RNA fast isolation kit (Bioteke, China) according to the manufacturer’s protocols. Then cDNA was generated using reverse transcriptase M-MLV (Takara, Japan). RT-PCR was performed using the SYBR green (Bioteke). The expressions of HSP110 and YAP were quantified using β-actin as an internal control. The PCR primers as following: human HSP110: sense 5′- ATAGGCCGCTTTGTAGT -3′, anti-sense 5′- CCATAGATGCCGTAGAG -3′, human YAP: sense 5′- AAGGCTTGACCCTCGTTT -3′, anti-sense 5′- TTGCTGTGCTGGGATTGA -3′, human β-actin: sense 5′- GGCACCCAGCACAATGAA -3′, anti-sense 5′- TAGAAGCATTTGCGGTGG -3′, mouse HSP110: sense 5′- AATGGTGGCGTGGGAATA -3′, anti-sense 5′- GGATGGGACTGAGATGAC -3′, mouse β-actin: sense 5′- CTGTGCCCATCTACGAGGGCTAT -3′, anti-sense 5′- TTTGATGTCACGCACGATTTCC -3′.

### Western blot

The total protein from tissues and cultured HPASMCs were isolated using cell lysis buffer for Western and IP kit (Beyotime, China) and the nuclear protein and cytoplasmic protein were extracted using nuclear and cytoplasmic protein extraction kit (Beyotime) according to the manufacturer’s protocols. Protein concentrations were determined using the BCA protein assay kit (Beyotime). Proteins were loaded and separated in SDS-PAGE (8, 10 and 15%) and immunoblotted with antibodies including HSP110 (Affinity, USA), LC3 (Abclonal, China), Beclin1 (Abclonal), ATG5 (Abclonal), ATG7 (Abclonal), p62 (Abclonal), p-YAP (Affinity), YAP (Santa cruz, USA), p-TAZ (Affinity), TAZ (Proteintech, China), TEAD4 (Abclonal), β-actin (Santa cruz) and histone H3 (Abgent, USA) overnight at 4 °C. Signal intensities of protein band were developed using ECL chemiluminescence kit (Beyotime) and the band densities were measured using gel imaging analysis system (LiuYi, China). The relative protein levels were calculated after normalization with β-action and compared to the appropriate control group.

### Immunofluorescence

The section (5 µm) from lung tissues were washed with PBS and separately co-incubated with HSP110 (Affinity) and YAP (Proteintech), HSP110 (Affinity) and alpha smooth muscle actin (α-SMA) (Affinity), YAP (Proteintech) and α-SMA (Abclonal), proliferating cell nuclear antigen (PCNA) (Abclonal) and α-SMA (Affinity), and Beclin1 (Abclonal) and α-SMA (Affinity) antibodies overnight at 4 °C. After washing, the sections were subject to second antibodies Cy3-labeled goat anti-rabbit IgG (Beyotime; Invitrogen, USA) and FITC-labeled goat anti-mouse IgG or anti-rabbit IgG (Beyotime; Abcam, UK) incubation at room temperature for 1.5 h. For cell immunofluorescence staining, fixed glass coverslips with cells were incubated with primary antibodies α-SMA (Affinity), Beclin1 (Abclonal) and YAP (Proteintech) overnight at 4 °C. Then the cells were subsequently incubated with fluorescent secondary antibodies Cy3-labeled goat anti-mouse IgG or anti-rabbit IgG (Beyotime) at room temperature for 1 h. The sections and coverslips then were incubated with DAPI (Aladdin, China) for counterstain and pictures were taken with fluorescence microscope (Olympus).

### Co-immunoprecipitation (Co-IP)

To verify the potential direct interaction between HSP110 and YAP, HEK293T cells were co-transfected with pECMV-3-FLAG-N-HSP110 (HSP110-Flag) alone or together with PCMV-MYC-YAP (YAP-Myc) for 48 h. The potential interaction between HSP110 and YAP was precipitated using anti-Myc (IP, Abclonal) and detected using anti-Flag (Abclonal) and anti-Myc (Abclonal) by western blot. Moreover, the interaction between YAP and TEAD4 in HPAMSCs was verified using anti-YAP (IP, Santa cruz) and the precipitates were detected by western blot using anti-TEAD4 (abclonal).

### Luciferase assay and chromatin immunoprecipitation (ChIP)

The HEK293T cells were seeded in 12-well plate and transiently transfected with PGL3-basic luciferase reporter vector harboring different human HSP110 promoter region fragments (− 2000/ + 20), (− 1000/ + 20) and (− 500/ + 20) with or without pcDNA3.1-TEAD4 plasmid (GenScript, China) using lipofectamine 3000 transfection reagent (Invitrogen) for 48 h. Empty vector pcDNA3.1 was used as a control. Luciferase activity was determined by a dual-luciferase reporter assay system (KeyGen Biotech, China). To verify the binding between TEAD4 and HSP110 promoter in HPAMSCs, the ChIP assay was performed using chromatin immunoprecipitation (ChIP) Kit (Wanleibio, China) according to the instructions. After hypnotic induction, the sonicated extracts of HPAMSCs were performed ChIP assay using anti-IgG or anti-TEAD4 antibody. After DNA purification, samples were performed PCR with the primers including three predicted TEAD4-binding sites (binding site 1: − 1952/− 1943, 5′- CAAATTCCTC -3′; binding site 2: − 1259/− 1250, 5′- GGAATTCCTC -3′; binding site 3: − 57/− 48, 5′- AACTTTCCAG -3′) on HSP110 promoter. The PCR primers as following: HSP110 promoter 1: sense 5′- AGTCGAGGAAACAGAGGG -3′, anti-sense 5′- CCTGGAGTCAGACATCATGGGCTTG -3′, HSP110 promoter 2: sense 5′- CATGTTAAGTAAGCCACC -3′, anti-sense 5′- CTTCTGATCCCTGAGTTT -3′, and HSP110 promoter 3: sense 5′- GAGAAGAAGGAAGCGGAAGTG -3′, anti-sense 5′- CCTCAGCCTTATGTATCGCACT -3′. Agarose gel electrophoresis was used for PCR products analysis.

### Pull-down assay

To confirm the binding between TEAD4 and HSP110 promoter in HPASMCs under hypoxia, pull-down binding assay was performed using one of the TEAD4-binding sites (binding site 3). After hypoxic induction, the nuclear extract was prepared from HPASMCs and incubated with DNA beads with biotinylated-HSP110 DNA probe harboring inverted repeat sequences of TEAD4-binding site 3 (5′- CTGGAAAGTT -3′). After elution, the protein content of TEAD4 was analyzed by western blot.

### Statistical analysis

Data analysis was performed using GraphPad Prism version 8.0. Data are plotted as means ± standard deviation. Two-tailed unpaired student’s t-test was used for statistical significance between two groups. For more than two groups, one- or two-way ANOVA was conducted. p < 0.05 was considered statistically significant.

## Results

### HSP110 is upregulated in lung tissues of the hypoxia-induced PH mice

**Fig. 1:**
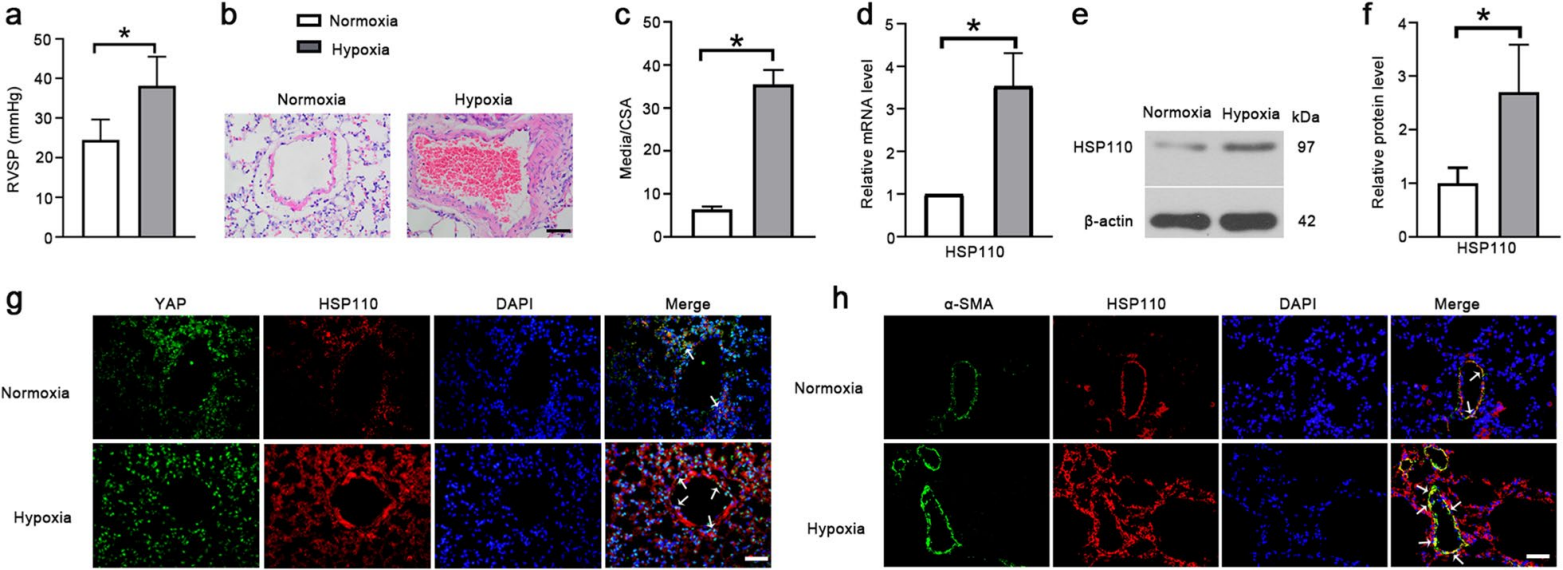
HSP110 is upregulated in lung tissues of the hypoxia-induced PH in mice. **a** The RVSP of the normoxia and hypoxia groups in mice (N = 8). **b** H&E staining of pulmonary arteries in lung tissues in normoxia and hypoxia group (N = 8). Scale bars, 50 μm. **c** Quantification of ratio of pulmonary arterial medial thickness to total vessel size (media/CSA) (N = 8). Relative mRNA level (**d**) and protein level (**e**, **f**) of HSP110 in pulmonary arteries (N = 8). **g** Double immunofluorescence staining of YAP (green) and HSP110 (red) in pulmonary arteries (N = 8). Scale bars, 50 μm. White arrows pointed to HSP110 and YAP double-positive cells. **h** Double immunofluorescence staining of α-SMA (green) and HSP110 (red) in pulmonary arteries (N = 8). Scale bars, 50 μm. White arrows pointed to HSP110 and α-SMA double-positive cells. Data are means ± SD from 8 mice per group. *p < 0.05

The RVSP was measured after a 4-week exposure to chronic hypoxia, the results in Fig. 1a showed that mice from hypoxia group developed a significant elevation in RVSP compared with normoxia mice. Pulmonary vascular morphological changes were observed by H&E staining. The results showed that hypoxia increased the pulmonary arterial medial wall thickness compared with the normoxia group (Fig. 1b, c). Next, we detected the expression of HSP110 in the pulmonary arteries. Hypoxia significantly increased the mRNA and protein levels of HSP110 compared to mice from normoxia group (Fig. 1d–f). Furthermore, the results in Fig. 1g showed that the HSP110 and YAP double-positive cells were increased in hypoxia group compared to normoxia group. To verify our hypotheses that HSP110 might have an important role in abnormal proliferation of PASMCs, double fluorescence staining of HSP110 and α-SMA were performed. The results displayed that HSP110 had a higher expression level in PASMCs in mice exposed to hypoxia than mice exposed to normoxia (Fig. 1h). The results suggest that HSP110 is highly expressed in pulmonary artery in hypoxia-induced PH mice and it might have important functions on PASMCs through YAP activation.

### HSP110 knockdown attenuates the development of PH induced by hypoxia

**Fig. 2:**
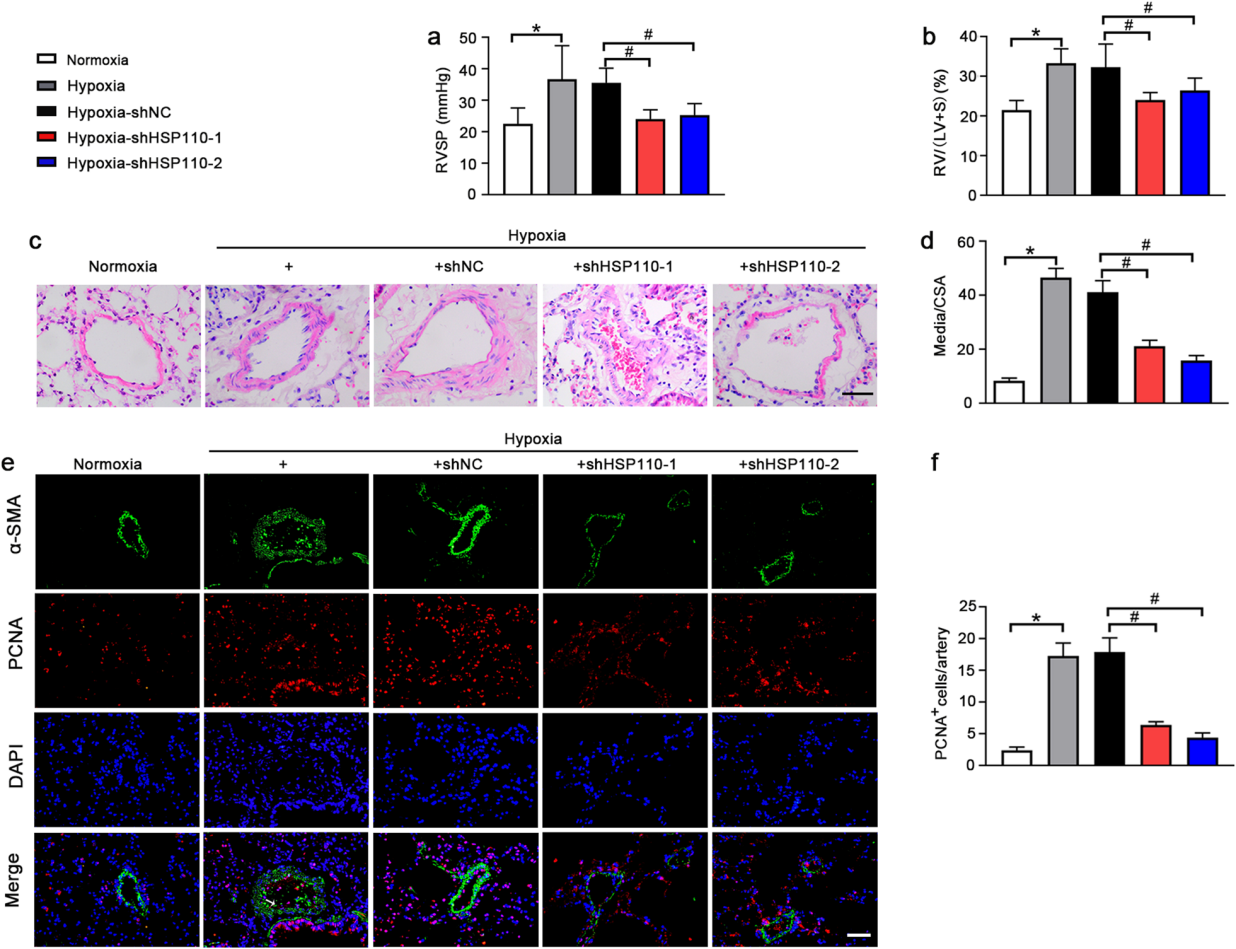
HSP110 knockdown attenuates the development of PAH induced by hypoxia. The RVSP (**a**) and RV/(LV + S) ratio (**b**) of the normoxia and hypoxia groups received with adeno-associated virus-mediated shHSP110 or shNC (N = 8). **c** H&E staining of pulmonary arteries in lung tissues in each group (N = 8). Scale bars, 50 μm. **d** Quantification of ratio of pulmonary arterial medial thickness to total vessel size (media/CSA) in each group (N = 8). **e** Double immunofluorescence staining of α-SMA (green) and PCNA (red) in pulmonary arteries in each group (N = 8). Scale bars, 50 μm. **f** Quantification of PCNA ^+^ cells in pulmonary arteries (N = 8). Data are means ± SD from 8 mice per group. *p < 0.05 compared to the normoxia group. ^#^p < 0.05 compared to the hypoxia-shNC group

To evaluate the effects of HSP110 on hypoxia-induced PH, adeno-associated virus carring the shHSP110-1 and shHSP110-2 were used to silence HSP110 expression in PH mice. After 4 weeks, the RVSP was remarkedly decreased in shHSP110-injected mice under hypoxia condition (Fig. 2a). In addition, the ratios of RV/(LV + S) were increased in hypoxia and hypoxia-shNC groups and knockdown of HSP110 dramatically reduced the increased RV/(LV + S) ratio (Fig. 2b). Furthermore, H&E staining for pulmonary arteries in Fig. 2c, d displayed that knockdown of HSP110 reduced the increases of pulmonary arterial medial wall thickness in PH mice. Knockdown of HSP110 inhibited the abnormal proliferation of cells in pulmonary artery by decreasing the PCNA expression (Fig. 2e, f). The results indicate that knockdown of HSP110 alleviates the development of hypoxia-induced PH in mice.

### HSP110 knockdown inhibits hypoxia-induced autophagy and YAP/TAZ-TEAD4 activity in mice

**Fig. 3:**
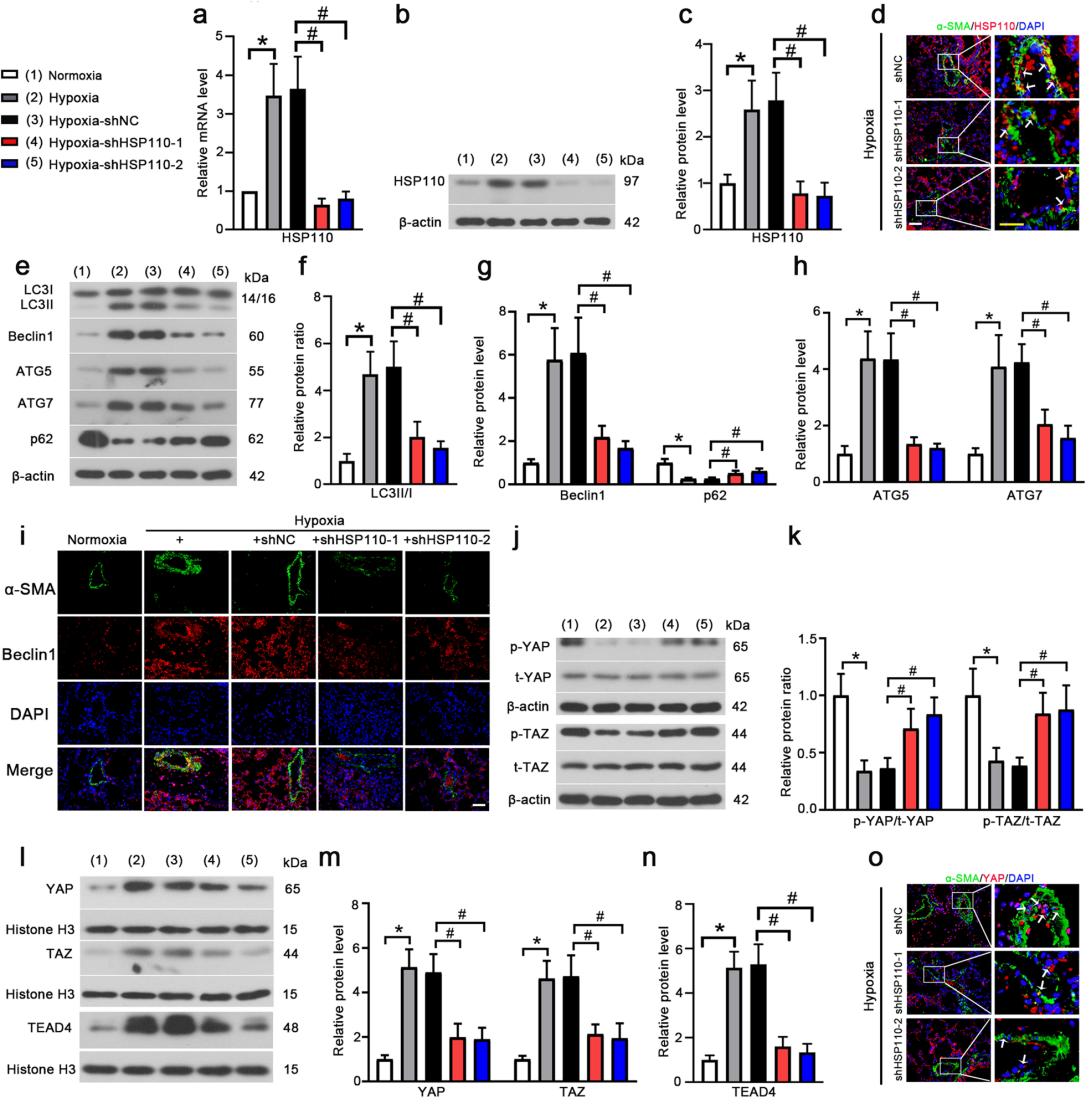
Knockdown of HSP110 inhibits hypoxia-induced autophagy and YAP/TAZ-TEAD4 activity in mice. Relative mRNA level (**a**) and protein level (**b**, **c**) of HSP110 pulmonary arteries in lung tissues in each group (N = 8). **d** Double immunofluorescence staining of α-SMA (green) and HSP110 (red) in pulmonary arteries (N = 8). White scale bars, 50 μm; Yellow scale bars, 25 μm. White arrows pointed to α-SMA and HSP110 double-positive cells. **e** Protein levels of LC3II, LC3I, Beclin1, ATG5, ATG7 and p62 in pulmonary arteries (N = 8). **f**–**h** Quantitative analysis of relative protein ratio of LC3-II/I and relative protein level of Beclin1, p62, ATG5 and ATG7 (N = 8). **i** Double immunofluorescence staining of α-SMA (green) and Beclin 1 (red) in pulmonary arteries (N = 8). Scale bars, 50 μm. **j** Protein levels of p-YAP, YAP, p-TAZ and TAZ in pulmonary arteries (N = 8). **k** Quantitative analysis of relative protein ratio of p-YAP/t-YAP and p-TAZ/t-TAZ (N = 8). **l**–**n** Nuclear protein levels of YAP, TAZ and TEAD4 and quantitative analysis of relative protein level of nuclear YAP, TAZ and TEAD4 (N = 8). **o** Double immunofluorescence staining of α-SMA (green) and YAP (red) in pulmonary arteries (N = 8). White scale bars, 50 μm; Yellow scale bars, 25 μm. White arrows pointed to α-SMA and YAP double-positive cells. Data are means ± SD from 8 mice per group. *p < 0.05 compared to the normoxia group. ^#^p < 0.05 compared to the hypoxia-shNC group

It’s wellknown that hypoxia could induce autophagy of PASMCs, which leads to promote the proliferation of PASMCs and increase pulmonary arterial wall thickness. In this work, we investigated whether HSP110 promotes hypoxia-induced autophagy in PASMCs in PH mice. Firstly, we confirmed the mRNA and protein expression of HSP110 were downregulated in lung tissue in shHSP110-1 and shHSP110-2 injected mice compared to shNC injected mice (Fig. 3a–c). Moreover, the expression of HSP110 was notably decreased in shHSP110 groups compared to shNC group in α-SMA-positive PASMCs (Fig. 3d). The occurrence of autophagy was determined by the increased LC3II/I, beclin1, ATG5 and ATG7 and the decreased p62 in the hypoxia-induced PH mice. More importantly, knockdown of HSP110 inhibited the LC3II/I, beclin1, ATG5 and ATG7 and elevated p62 expression compared to shNC group (Fig. 3e–h). Additionally, knockdown of HSP110 reduced the beclin1 expression in α-SMA-positive cells in pulmonary artery (Fig. 3i). For mechanism study, we assessed the activation of YAP/TAZ-TEAD4 pathway. The results displayed that knockdown of HSP110 inhibited the activation of YAP/TAZ-TEAD4 pathway by increasing the phosphorylated YAP and TAZ and decreasing nuclear YAP, TAZ and TEAD4 (Fig. 3j–n). Moreover, YAP nuclear translocation in α-SMA-positive PASMCs was notably decreased in mice that received shHSP110 compared to shNC (Fig. 3o). The results indicate that knockdown of HSP110 inhibits hypoxia-induced autophagy and inactivates YAP/TAZ-TEAD4 pathway in pulmonary artery in PH mice.

### HSP110 affects hypoxia-induced proliferation, migration and autophagy in HPASMCs

**Fig. 4:**
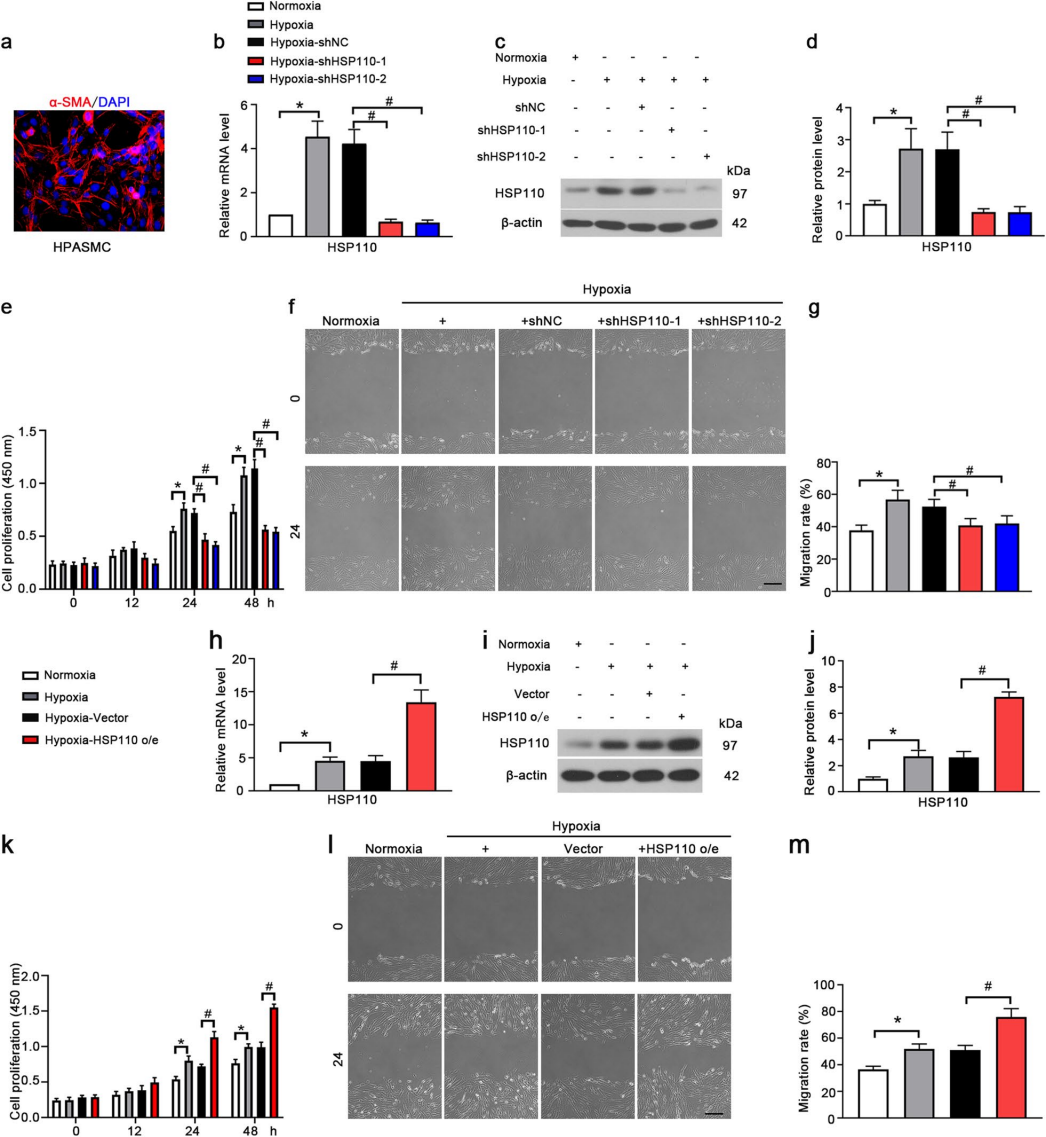
HSP110 affects hypoxia-induced proliferation and migration in HPASMCs. **a** The identification of HPASMCs by α-SMA staining (N = 1). Scale bars, 50 μm. Relative mRNA level (**b**) and protein level (**c**, **d**) of HSP110 in HPASMCs received with shHSP110 or shNC after 24 h hypoxic exposure (N = 3). **e** Cell proliferation of HPASMCs in each group after different time hypoxic exposure (N = 3). Wound healing assay (**f**) and quantification of migration ratio (**g**) of HPASMCs in each group (N = 3). Scale bars, 200 μm. Data are means ± SD from 3 biological replicates. In **b**, **d**, **e** and **g**, *p < 0.05 compared to the normoxia group. ^#^p < 0.05 compared to the hypoxia-shNC group. Relative mRNA level (**h**) and protein level (**i**, **j**) of HSP110 in HPASMCs received with adenovirus vector carring HSP110 cDNA or control vector after 24 h hypoxic exposure (N = 3). **k** Cell proliferation of HPASMCs in each group after different time hypoxic exposure (N = 3). Wound healing assay (**l**) and quantification of migration ratio (**m**) of HPASMCs in each group (N = 3). Scale bars, 200 μm. Data are means ± SD from 3 biological replicates. In **h**, **j**, **k** and **m**, *p < 0.05 compared to the normoxia group. ^#^p < 0.05 compared to the hypoxia-Vecotor group

The HPASMCs in vitro were identified using SMC marker α-SMA (Fig. 4a). Then HPASMCs were infected with adenoviral vector packaging with shHSP110-1 and shHSP110-2 for 48 h and subsequently subjected to hypoxic induction. The results showed that hypoxia increased the expression of HSP110, and shHSP110-1 and shHSP110-2 significantly decreased HSP110 expression compared to shNC (Fig. 4b–d). The effects of HSP110 on hypoxia-induced proliferation and migration of HPASMCs were evaluated. Hypoxia promoted the proliferation and migration of HPASMCs, and knockdown of HSP110 significantly reduced that increases of proliferation and migration of HPASMCs (Fig. 4e–g). The opposite results were obtained in HSP110 overexpressing HPASMCs. Adenovirus carring HSP110 overexpression vector increased the HSP110 mRNA and protein expression in HPASMCs (Fig. 4h–j). Overexpression of HSP110 further increased the cell proliferation and migration of HPASMCs under hypoxia (Fig. 4k–m). The results indicated that HSP110 promoted hypoxia-induced proliferation and migration of HPASMCs.

**Fig. 5:**
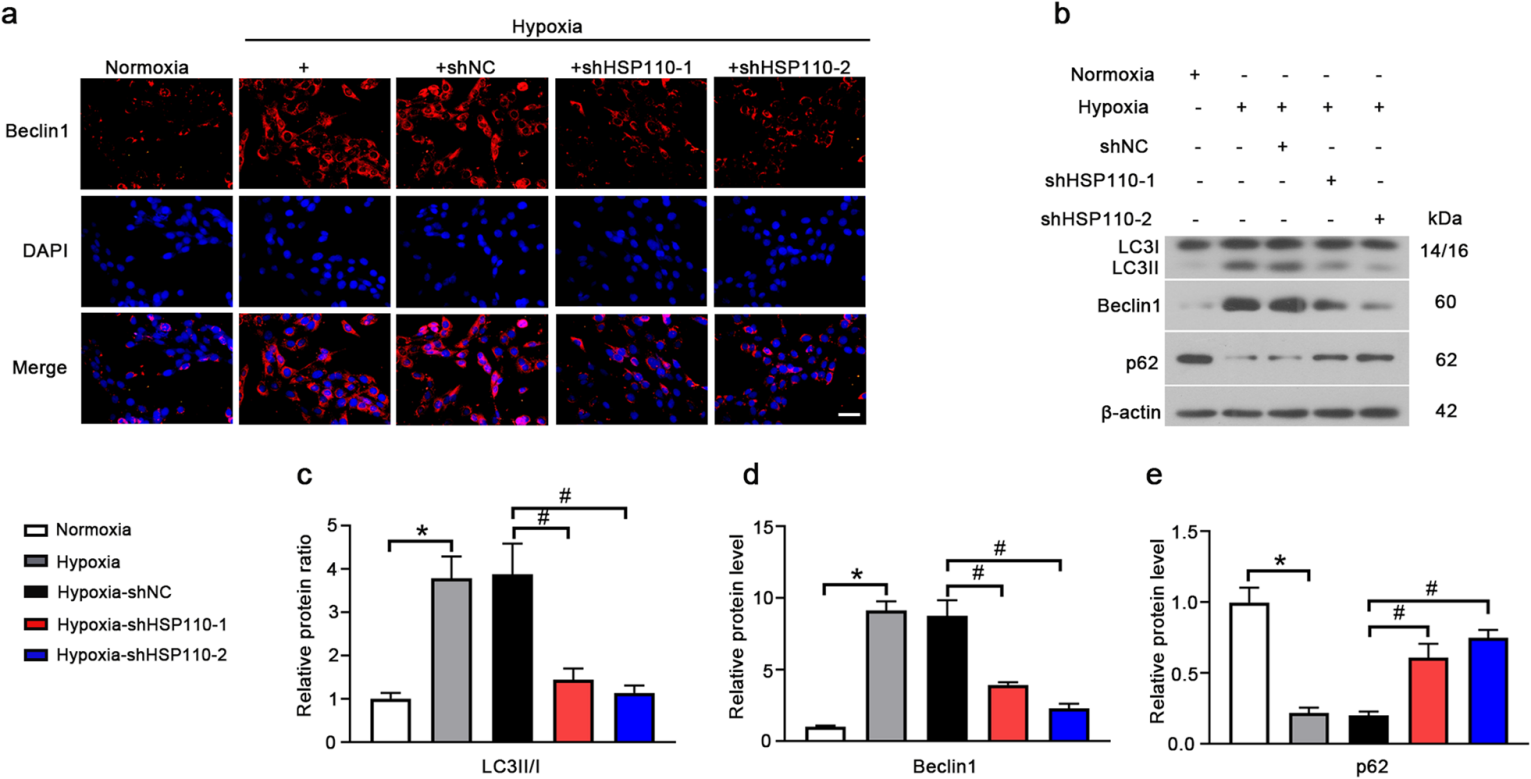
Knockdown of HSP110 inhibits hypoxia-induced autophagy in HPASMCs. **a** Immunofluorescence staining of Beclin 1 (red) in HPASMCs in each group. Scale bars, 50 μm. **b** Protein levels of LC3I, LC3II, Beclin 1 and p62 in HPASMCs. **c**–**e** Quantitative analysis of relative protein ratio of LC3-II/I and relative protein level of Beclin1 and p62. Data are means ± SD from 3 biological replicates. *p < 0.05 compared to the normoxia group. ^#^p < 0.05 compared to the hypoxia-shNC group

The effects of HSP110 on hypoxia-induced autophagy were determined by detecting the expression of autophagy-related proteins. Immunofluorescence staining showed that the beclin1 expression was increased under hypoxia and decreased in shHSP110 treated cells (Fig. 5a). Moreover, knockdown of HSP110 inhibited LC3II/I and beclin1 and elevated p62 compared to shNC group (Fig. 5b–e). The results suggest that HSP110 could positively affect hypoxia-induced autophagy in HPASMCs.

### HSP110 affects HPASMCs through YAP/TAZ-TEAD4 pathway

**Fig. 6:**
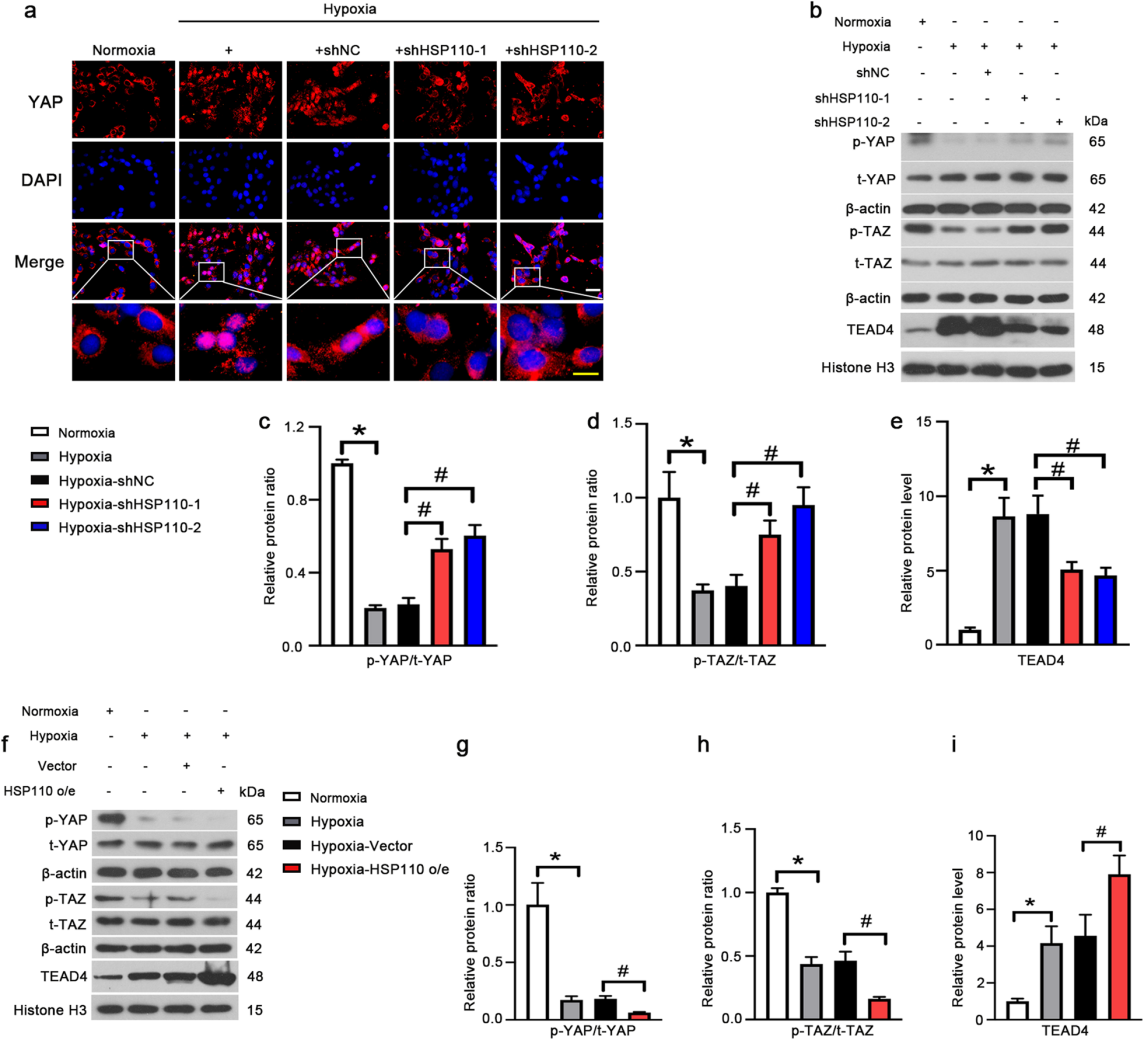
HSP110 activates YAP/TAZ-TEAD4 pathway in HPASMCs. **a** Immunofluorescence staining of YAP (red) in HPASMCs received with shHSP110 or shNC after 24 h hypoxic exposure in each group. White scale bars, 50 μm; Yellow scale bars, 25 μm. **b**–**e** Protein level p-YAP, YAP, p-TAZ, TAZ and nuclear TEAD4 and quantitative analysis of relative protein ratio of p-YAP/t-YAP and p-TAZ/t-TAZ and relative protein level of TEAD4. Data are means ± SD from 3 biological replicates. In **c**–**e**, *p < 0.05 compared to the normoxia group. ^#^p < 0.05 compared to the hypoxia-shNC group. f-i HPASMCs received with adenovirus vector carring HSP110 cDNA or control vector after 24 h hypoxic exposure. Protein levels p-YAP, t-YAP, p-TAZ, t-TAZ and nuclear TEAD4 and quantitative analysis of relative protein ratio of p-YAP/t-YAP, p-TAZ/t-TAZ and relative protein level of nuclear TEAD4. Data are means ± SD from 3 biological replicates. In **g**–**i**, *p < 0.05 compared to the normoxia group. ^#^p < 0.05 compared to the hypoxia-Vector group

**Fig. 7:**
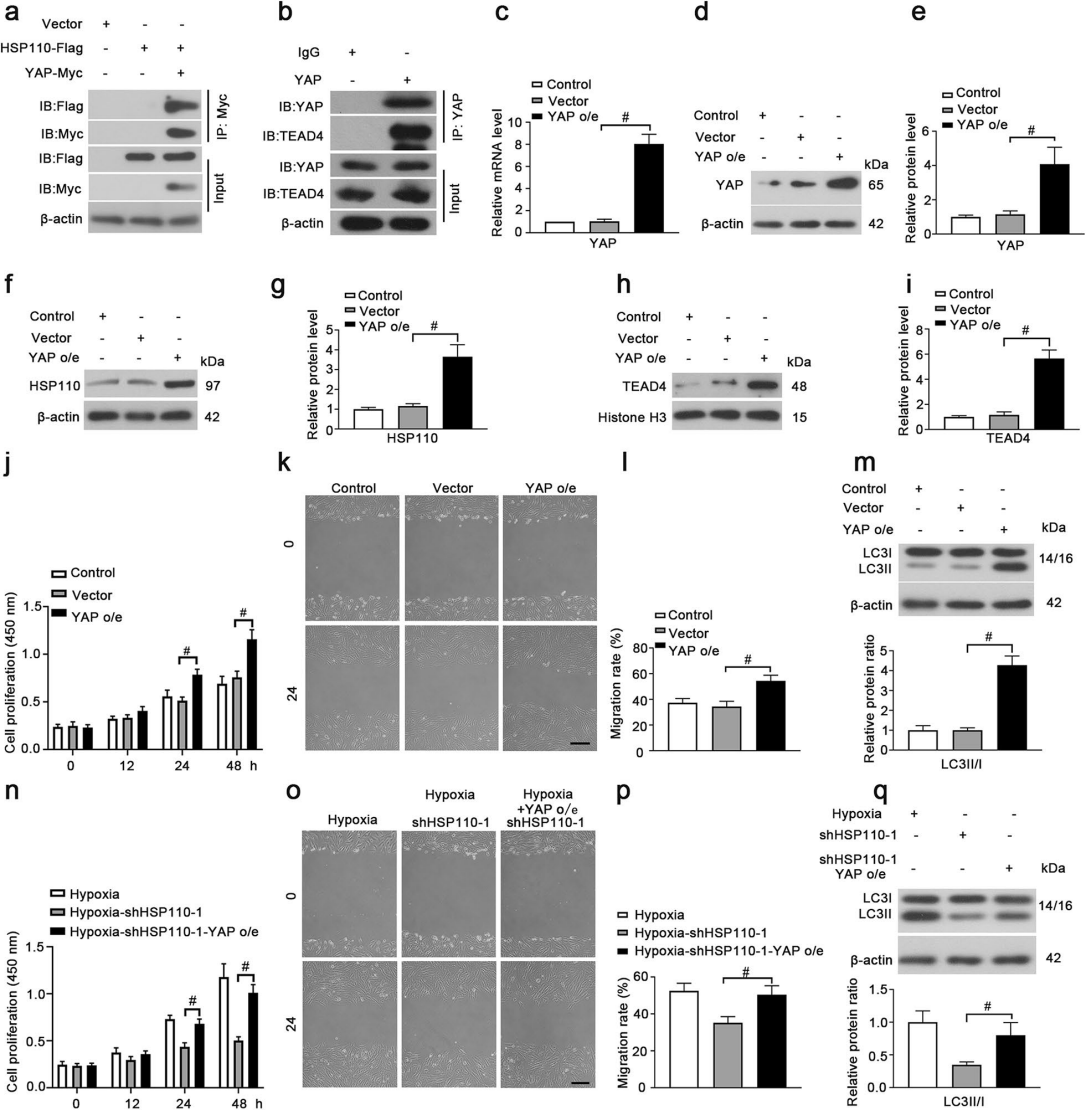
HSP110 promotes cell proliferation, migration and autophagy through YAP/TAZ-TEAD4 pathway. **a** HEK293T cells were transfected with the plasmids for overexpressing HSP110-Flag and/or YAP-Myc for 48 h. The cell lysates were immunoprecipitated using anti-Myc antibody. The protein levels of HSP110 and YAP in precipitates and input were detected. **b** The cell lysates from HPASMCs were immunoprecipitated using anti-YAP antibody. The interaction of YAP and TEAD4 was detected western blot. HPASMCs were infected with adenovirus vector overexpressing YAP (YAP o/e) or empty vector for 48 h, relative mRNA level (**c**) and protein level (**d**, **e**) of YAP in HPASMCs in each group. **f**–**i** Protein levels HSP110 and nuclear TEAD4 and quantitative analysis of relative protein level of HSP110 and TEAD4. **j** Cell proliferation of YAP overexpessing HPASMCs after different time hypoxic exposure. Wound healing assay (**k**) and quantification of migration ratio (**l**) of HPASMCs in each group. Scale bars, 200 μm. **m** Protein levels of LC3I and LC3II and quantitative analysis of relative protein ratio of LC3-II/I in HPASMCs. **n** HPASMCs were co-infected with YAP o/e and shHSP110-1 for 48 h, the cell proliferation of HPASMCs in each group after hypoxic exposure. Wound healing assay (**o**) and quantification of migration ratio (**p**) of HPASMCs in each group. Scale bars, 200 μm. q Protein levels of LC3I and LC3II and quantitative analysis of relative protein ratio of LC3-II/I in HPASMCs. Data are means ± SD from 3 biological replicates. ^#^p < 0.05 compared to the vector or hypoxia-shHSP110-1 group

In this study, we investigated whether HSP110 plays its role in HPASMCs through the YAP/TAZ-TEAD4 pathway. Hypoxia inhibited phosphorylation of YAP/TAZ and promoted the YAP nuclear translocation and nuclear TEAD4 expression, and the effects could be rescued by HSP110 knockdown (Fig. 6a-e). Additionally, overexpression of HSP110 further induced the YAP/TAZ-TEAD4 activation (Fig. 6f–i). We experimentally verified the interaction between YAP and HSP110 and the interaction between YAP and TEAD4 (Fig. 7a, b). Next, we wondered whether overexpression of YAP could partially reverse the effects of HSP110 on HPASMCs. Adenovirus carring YAP overexpression vector was infected into HPASMCs and increased YAP mRNA and protein expression (Fig. 7c–e). Interestingly, overexpression of YAP significantly increased the HSP110 expression and nuclear TEAD4 expression (Fig. 7f–i). More importantly, overexpression of YAP not only increased the proliferation, migration and autophagy of HPASMCs (Fig. 7j–m), but also reduced the effects of HSP110 knockdown on HPASMCs under hypoxia condition (Fig. 7n–q). The results indicated that HSP110 promoted hypoxia-induced proliferation, migration and autophagy of HPASMCs through YAP/TAZ-TEAD4 pathway.

### TEAD4 promotes HSP110 transcription by binding to HSP110 promoter

**Fig. 8:**
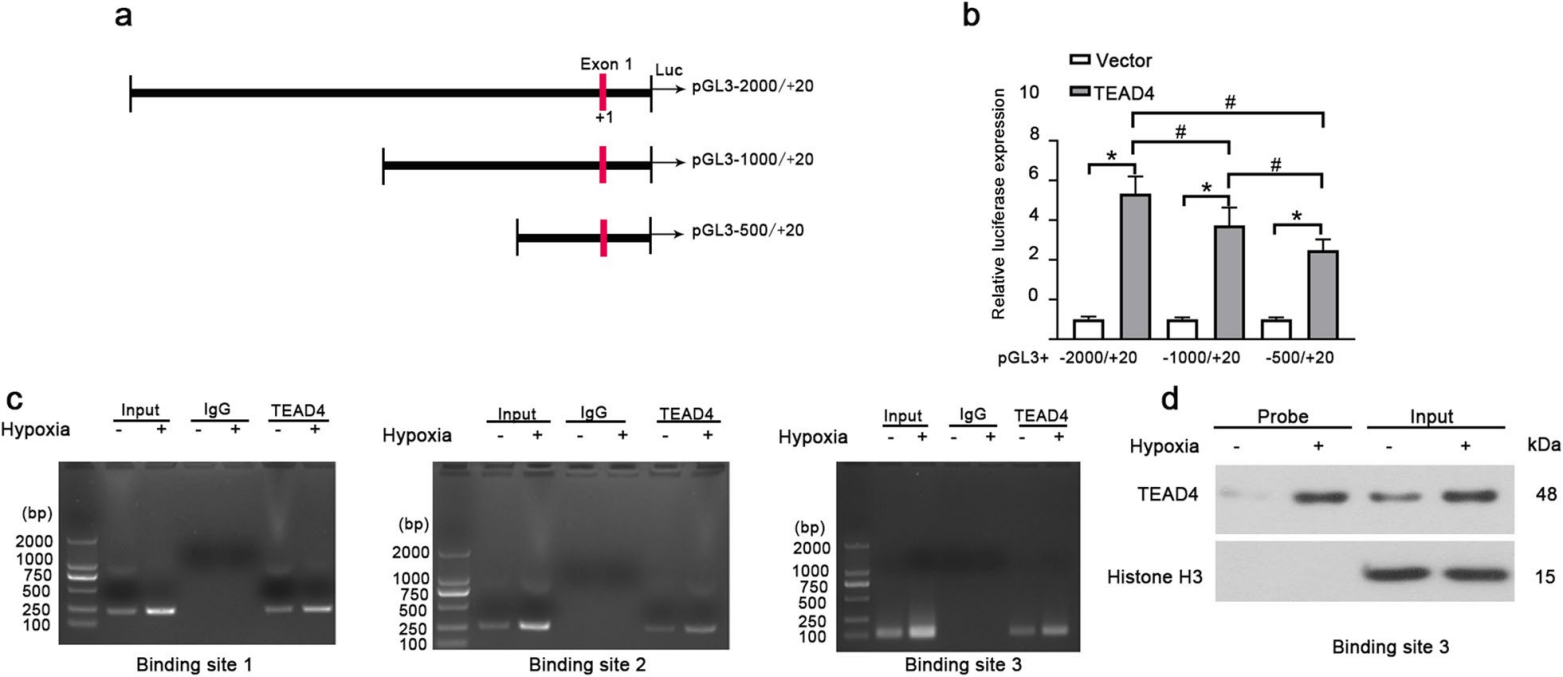
TEAD4 promotes HSP110 transcription by binding to HSP110 promoter. **a** Schematic representation of the pGL3-basic-HSP110 promoter reporter constructs used in this study. The HSP110 promoter (− 2000/ + 20) and sequential deletion of the HSP110 promoter shorter fragments (− 1000/ + 20, − 500/ + 20) were cloned into the pGL3-basic luciferase vector. **b** Constructs with the HSP110 promoter fragments were co-transfected with overexpression vector containing TEAD4 or empty vector into HEK293 cells. The luciferase activity was analyzed. Data are means ± SD from 3 biological replicates. *p < 0.05 compared to vector, ^#^p < 0.05 compared to HSP110 promoter fragments − 2000/ + 20 or − 1000/ + 20 under TEAD4 overexpression. **c** The chromatin immunoprecipitation of the HSP110 promoter with anti-TEAD4 or anti-IgG antibodies was performed using HPASMCs after hypoxic exposure. The sequences containing the TEAD4-binding sites on HSP110 promoter were amplified by PCR and detected by agarose gel. **d** The nuclear extract was prepared from HPASMCs after hypoxic exposure and biotinylated-HSP110 DNA probe specific to TEAD4-binding site 3 was used for pull-down assay. The protein level of TEAD4 was detected

TEAD4 is one of transcription factors from TEAD family and it could regulate transcriptions of many target genes. Therefore, we wondered whether TEAD4 could regulate HSP110 transcription. We predicted TEAD4 target sites on HSP110 promoter using JASPAR database. HEK293 cells were separately transfected with a series of truncations of HSP110 promoter (− 2000/ + 20, − 1000/ + 20 and − 500/ + 20) coupled luciferase reporter and co-transfected with plasmid expressing TEAD4 (Fig. 8a). HSP110 promoter activity was enhanced by TEAD4 and significantly reduced by deletion of the HSP110 promoter sequence (Fig. 8b). The results indicate that TEAD4 could regulate the HSP110 transcription by binding HSP110 promoter. To corroborate the data from luciferase assay and verify whether hypoxia affects the interaction, the physical interaction of TEAD4 with the HSP110 promoter was examined by ChIP assay in HPASMCs under hypoxia condition. As shown in Fig. 8c, TEAD4 specifically bound HSP110 promoter on TEAD4 target sites and hypoxia promoted the binding ability between TEAD4 and HSP110 promoter. Moreover, oligonucleotide probe harboring inverted repeat sequences of TEAD4-binding site 3 verified the interaction between TEAD4 and HSP110 promoter and hypoxia promoted the interaction (Fig. 8d). The results indicate that TEAD4 promotes HSP110 transcription by binding to HSP110 promoter  9).

## Discussion

**Fig. 9:**
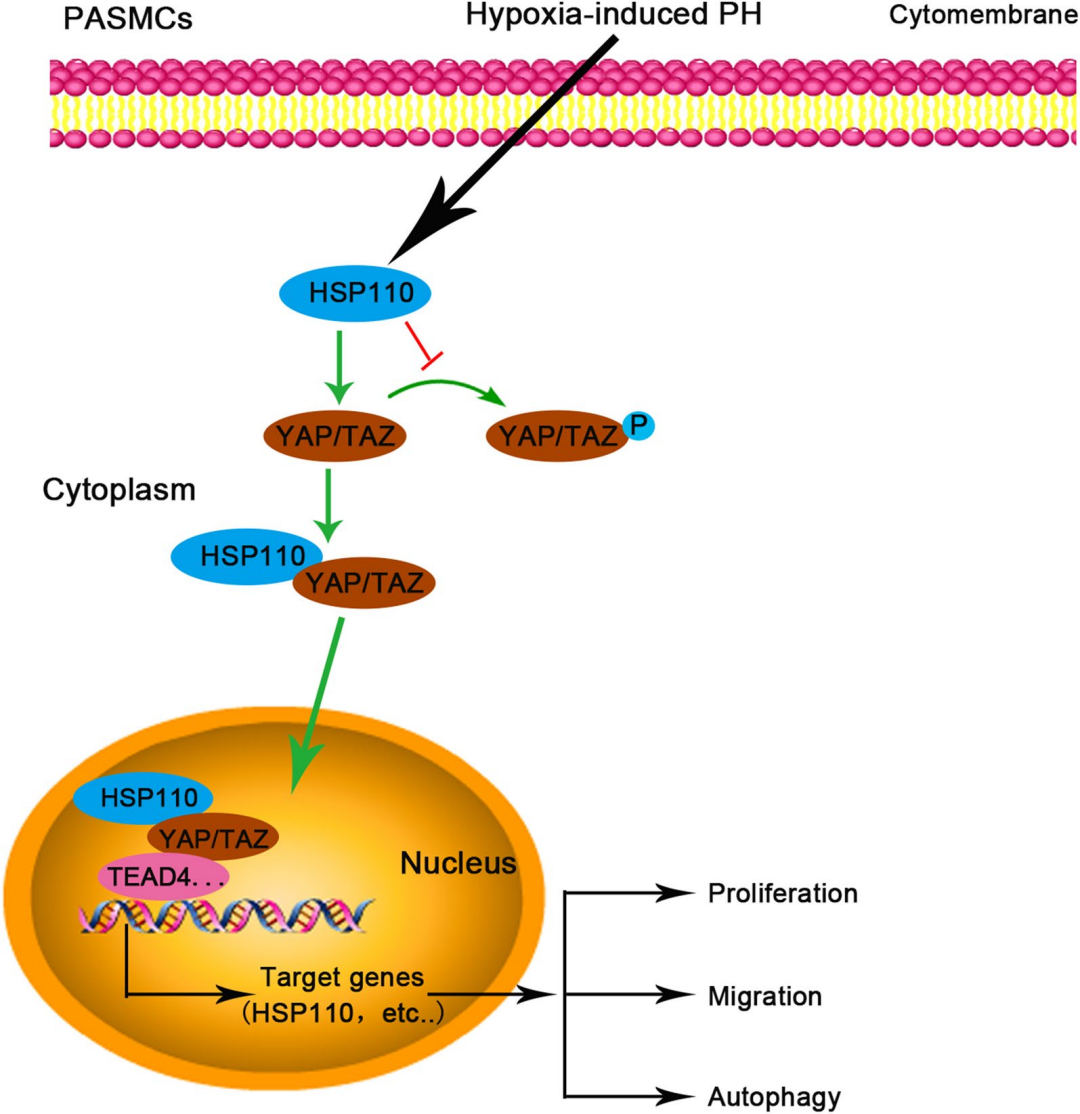
The molecular mechanism of HSP110 in PH. In PH, the expression of HSP110 is increased in PASMCs, which leads to more HSP110 interacting with YAP and inhibiting YAP/TAZ phosphorylation, and thus promoting YAP/TAZ nuclear translocation. In nucleus, YAP/TAZ binds to transcription factor such as TEAD4 to regulate transcription of target genes involved in cellular proliferation, migration and autophagy. Moreover, TEAD4 could promote the transcriptional activity of HSP110 promoter by binding to the HSP110 promoter in PASMCs

In this work, we demonstrated HSP110 might play important roles in hypoxia-induced PH in mice model in vivo and hypoxia-induced HPASMCs proliferation, migration and autophagy in vitro. HSP110 was upregulated in pulmonary tissues of mice and HPASMCs after hypoxia exposure. Knockdown of HSP110 by shRNA significantly alleviated the hypoxia-induced PH and inhibited proliferation, migration and autophagy of PASMCs under hypoxia compared to normoxia. More importantly, we proved that HSP110 functions in PASMCs might be through regulating YAP/TAZ-TEAD4 pathway and TEAD4 could regulate HSP110 transcription by binding HSP110 promoter (Fig. 9).

PH is a progressive and fatal disease which can be contributed by many pathogenesis including vasoconstriction, vascular remodeling and situ thromboses of small pulmonary arteries [31, 32]. Pulmonary vascular remodeling is the key feature of PH and associated with excessive proliferation/migration of PASMCs in the arterioles, which leads to increase of medial thickness and vascular resistance [31, 33]. Studies found that abnormal proliferation and migration of PASMCs have been found to increase the pulmonary artery medial thickness, pulmonary artery pressure, and ultimately lead to right heart hypertrophy [34–36]. In addition, autophagy has to be known as a housekeeping mechanism that maintains cellular homeostasis and promotes the cell survival when cells suffer under cellular stress such as hypoxia [7, 9, 37]. Numbers of studies have revealed that autophagy is activated in experimental PH model, and autophagy contributes to the proliferation and migration of PASMCs to aggravate PH in animal model [10, 38]. Our results demonstrated that HSP110 knockdown inhibited the proliferation of PASMCs and alleviated RVSP, arterial wall thickening, and right ventricular hypertrophy in vivo. To verify the findings in vivo, we found that HSP110 promoted the hypoxia-induced proliferation, migration and autophagy of HPASMCs in vitro.

The functions of HSP110 on cell proliferation through regulating other protein-related pathway have been reported. For instance, in activated B cell diffuse large B cell lymphoma (ABC-DLBCL), HSP110 promotes the proliferation of ABC-DLBCL cell lines by enhancing MyD88-mediated NF-κB activity [19]. Additionally, HSP110 activates phosphorylation of STAT3 and promote colorectal cancer growth [17]. Therefore, we tried to predict some new proteins that might interact and work with HSP110 in PASMCs. In our case, we predicted that YAP has a potential interaction with HSP110. It has proved that YAP promotes the proliferation and migration of PAVSMCs through inducing Notch3 expression or interacting with SMAD2 to play an important role in PH development [39, 40]. In addition, Fu et al. found that fibulin-5 promoted human airway smooth muscle cell proliferation and migration and increased YAP/TAZ expression [41]. YAP/TAZ could promote cell proliferation and migration of vascular smooth muscle cells (VSMCs) after vascular injury [42]. More importantly, YAP and TAZ are important for autophagosomal degradation in autophagy and there are evidences prove that YAP and TAZ could induce autophagy [29, 30, 43]. Therefore, it is very likely that the roles of HSP110 in PH are through regulating YAP/TAZ activity. It is wellknown that YAP and TAZ don’t have DNA-binding domain and they form a complex with the transcription factor like TEADs to promote the transcription of target genes [22, 44, 45]. In this work, we found that knockdown of HSP110 promoted phosphorylation of YAP/TAZ and inhibit nuclear translocation of YAP/TAZ in HPASMCs, it meant knockdown of HSP110 inhibited the YAP/TAZ transcriptional regulatory activity. Moreover, the expression of TEAD4 in nuclear was dramatically reduced in shHSP110-infected cells compared to shNC-infected cells under hypoxia. Conversely, overexpression of HSP110 increased YAP/TAZ-TEAD4 activity in HPASMCs. Co-IP assays demonstrated the interaction HSP110 could bind with YAP as predicted and the interaction between YAP and TEAD4 HPASMCs was confirmed. Furthermore, overexpression of YAP reversed the effects of HSP110 knockdown on proliferation, migration and autophagy in HPASMCs. The results indicate the functions of HSP110 in HPASMCs under hypoxia are related with YAP/TAZ-TEAD4 pathway.

TEAD4 is a key member of the TEAD family and it binds to different cofactors to regulate the transcription of target genes that involved in cellular cell functions [22]. TEAD4 plays an oncogenic role in many cancers including gastric cancer, hepatocellular carcinoma, lung adenocarcinoma and head neck squamous cell carcinoma by increasing cancer cell proliferation, migration and epithelial-mesenchymal transition (EMT) [46–48]. For example, Li et al. reported that YAP/TEAD4 interaction mediates transcriptional activity of KIF4A promoter by binding KIF4A promoter, which will promote cell growth of esophageal squamous cell carcinoma (ESCC) cells [49]. More importantly, DKK1 (dickkopf-1) increases ASMC proliferation and migration by upregulating ubiquitin-like containing PHD and RING finger domains 1 (UHRF1) expression through YAP-TEAD1/4 pathway [50]. Our results proved there were multiple TEAD4-binding sits on the HSP110 promoter. Moreover, luciferase assay verified that TEAD4 could promote the transcriptional activity of HSP110 promoter. Additionally, hypoxia promoted the binding ability between TEAD4 and HSP110 promoter in HPASMCs. It indicates that it’s possible that hypoxia enhances HSP110 expression partially through TEAD4 in HPASMCs, which will further promote cell proliferation and migration of HPASMCs and contribute to PH development.

In this study, we focused on the effects of HSP110 on the PAVSMCs but not other pulmonary vascular/immune cell types such adventitial fibroblasts, endothelial cells and macrophages. In addition, AAV2 is a common a delivery viral vector in AAV-based research and in clinical gene therapy for humans. However, AAV2-medicated HSP110 knockdown was not specific in PAVSMCs in PH mice in this study. Therefore, we used HPAVSMCs in vitro to verify our findings in vivo. Given our findings that HSP110 was upregulated not only in α-SMA positive cells and knockdown of HSP110 by AAV2-carring shHSP110 attenuated hypoxia-induced PH in mice, the functions of HSP110 in other human PH pulmonary cell types and experimental PH models warrant further investigation. Furthermore, the potential regulatory mechanism for regulating HSP110 expression in PAVSMCs is worthy of investigation.

## Conclusion

In summary, our findings suggest that knockdown of HSP110 alleviates hypoxia-induced PH in mice and inhibits proliferation, migration and autophagy of PASMCs, at least partially, through regulating YAP/TAZ-TEAD4 pathway, and the transcription of HSP110 could be regulated by TEAD4. Our findings might help to further understand the pathogenic mechanism in PH development.

## Data Availability

The datasets used and analyzed during the current study are available from the corresponding author on reasonable request.

## Abbreviations

ChIP: Chromatin immunoprecipitation
Co-IP: Co-immunoprecipitation
DKK1: Dickkopf-1
EMT: Epithelial-mesenchymal transition
ESCC: Esophageal squamous cell carcinoma
HUVECs: Human umbilical vein endothelial cells
HSP110: Heat shock protein-110
H&E: Hematoxylin and eosin
LPS: Lipopolysaccharide
LV: Left ventricular
Media/CSA: Pulmonary arterial medial thickness to total vessel size
NC: Negative control
PAH: Pulmonary arterial hypertension
PAECs: Pulmonary artery endothelial cells
PASMCs: Pulmonary artery smooth muscle cells
PCNA: Proliferating cell nuclear antigen
RT-PCR: Real-time PCR
RV: Right ventricle
RVSP: Right ventricle systolic pressure
S: Interventricular septum
STAT3: Signal transducer and activator of transcription 3
TAZ: Transcriptional co-activator with PDZ-binding motif
TEAD: Transcriptional enhanced associate domain
UHRF1: Ubiquitin-like containing PHD and RING finger domains 1
VSMCs: Vascular smooth muscle cells
YAP: Yes-associated protein
α-SMA: Alpha smooth muscle actin
